# Facilitating In-House Mobile App Development Within Psychiatric Outpatient Services for Patients Diagnosed With Borderline Personality Disorder: Rapid Application Development Approach

**DOI:** 10.2196/46928

**Published:** 2023-11-30

**Authors:** Ali Abbas Shaker, Stephen F Austin, Mie Sedoc Jørgensen, John Aasted Sørensen, Henrik Bechmann, Henriette E Kinnerup, Charlotte Juul Petersen, Ragnar Klein Olsen, Erik Simonsen

**Affiliations:** 1 Psychiatric Research Unit Psychiatric Department, Region Zealand Psychiatry Slagelse Denmark; 2 Department of Clinical Medicine, University of Copenhagen Copenhagen Denmark; 3 Department of Psychology Faculty of Health Sciences University of Southern Denmark Odense Denmark; 4 Research Unit: AI, Mathematics and Software Department of Engineering Technology and Didactics Technical University of Denmark Ballerup Denmark; 5 Mental Health Services South Psychiatry Region Zealand Maribo Denmark; 6 Mental Health Services South, Copenhagen University Hospital Psychiatry Region Zealand Vordingborg Denmark; 7 Mental Health Services East, Copenhagen University Hospital Psychiatry Region Zealand Roskilde Denmark

**Keywords:** software models, in-house development, psychiatric services, borderline personality disorder, mobile application, development, mental health, user design, design, psychiatric, implementation, innovation

## Abstract

**Background:**

Mobile app development within mental health is often time- and resource-consuming, challenging the development of mobile apps for psychiatry. There is a continuum of software development methods ranging from linear (waterfall model) to continuous adaption (Scrum). Rapid application development (RAD) is a model that so far has not been applied to psychiatric settings and may have some advantages over other models.

**Objective:**

This study aims to explore the utility of the RAD model in developing a mobile app for patients with borderline personality disorder (BPD) in a psychiatric outpatient setting.

**Methods:**

The 4 phases of the RAD model: (1) requirements planning, (2) user design, (3) construction, and (4) cutover, were applied to develop a mobile app within psychiatric outpatient services for patients diagnosed with BPD.

**Results:**

For the requirements planning phase, a short time frame was selected to minimize the time between product conceptualization and access within a clinical setting. Evidenced-based interactive content already developed was provided by current staff to enhance usability and trustworthiness. For the user design phase, activity with video themes and a discrete number of functions were used to improve the app functionality and graphical user interface. For the construction phase, close collaboration between clinicians, researchers, and software developers yielded a fully functional, in-house–developed app ready to be tested in clinical practice. For the cutover phase, the mobile app was tested successfully with a small number (n=5) of patients with a BPD.

**Conclusions:**

The RAD model could be meaningfully applied in a psychiatric setting to develop an app for BPD within a relatively short time period from conceptualization to implementation in the clinic. Short time frames and identifying a limited number of stakeholders with relevant skills in-house facilitated the use of this model. Despite some limitations, RAD could be a useful model in the development of apps for clinical populations to enable development and access to evidence-based technology.

## Introduction

### Overview

A growing evidence base suggests that mobile apps within the psychiatric field are a promising tool for patients seeking mental health–related information [1,2]. However, developing evidence-based mental health apps often requires cooperation between multiple stakeholders, that is, patients, clinicians, software developers, and project managers [3]. Thus, mobile app development is often time- and resource-consuming, leading to challenges in the development of apps for psychiatry.

There is a continuum of software development methods. At one extreme, it is assumed that the software development process is completely specified from the outset. One of the most basic models is the waterfall model [4]. The model is built up on sequential (linear) phases and includes starting with describing the requirements for the (1) app, (2) analysis, (3) design, (4) coding, (5) testing, and (6) maintenance of the app. The advantage of the waterfall model is the linear workflow where one finishes 1 phase and moves to the next. Unfortunately, this model is not suited for app development requiring ongoing adaptions [5,6]. For example, changes in demands from patients or clinicians require adjustments to the development process.

At the other extreme, it is assumed that the development process is carried out continuously, leading to constant system adaption to the patient or therapy system. The agile model “Scrum,” is an example of this type of model often used within large and complex software systems [7]. It requires multiple software developers, project managers, and Scrum masters and is generally an expensive and time-consuming software development model [8]. Scrum consists of the same phases as mentioned for the waterfall model, although it differs in the nonlinear workflow during the developing process, where sprints and iterations are needed to adapt to new or modified requirements.

A software development model that lies between these 2 models on the continuum is rapid application development (RAD) [9]. The main advantage of applying RAD for developing the mobile app is that it enables a short iteration time and fast adaptation to the complete end user path, including clinicians, software developers, and patients. To our knowledge, RAD has not been applied within a psychiatric outpatient setting before. The model has the potential within psychiatry to encourage clinicians to contribute to the app development process due to the minimal effort needed from clinicians during the development process. The RAD model also has the potential to increase the number of evidence-based mobile apps launched in the future at app markets (App Store and Google Play), which unfortunately are overrepresented with nonevidence-based mobile apps [10]. The RAD model can also deliver software solutions of high quality, with fast development, and low costs [9].

While psychotherapy can be effective for treating borderline personality disorder (BPD), waiting for treatment can be a problem, with patients often experiencing distress and disruption to care. Multiple studies have addressed the importance of continuity of care within mental health services. These studies highlight that continuity of care can improve several patient outcomes. First, continuity in care can increase patient satisfaction experienced during their encounters with mental health services. Second, the continuity of care also contributes to an improved patient-therapist alliance. Alliance is recognized as an important factor in psychotherapy outcomes [11]. Third, improved satisfaction and alliance can promote patient adherence to treatment.

Thus, the long waiting time for the initiation of psychotherapy that some patients with BPD experience can significantly impact and affect the continuity of care. Apps targeting patients with BPD waiting to commence psychotherapy have the potential to improve the continuity of care for these patients.

### Objective

This study aims to explore the utility of the RAD model in developing a mobile app for patients with BPD in a psychiatric outpatient setting.

## Methods

### Overview

The RAD method was selected as the software development model because it offers a selection of well-matching characteristics, both with respect to the time span of an app development step and high granular specification steps, carried out in close collaboration between the project participants [9,12]. Furthermore, due to the timeframe and limited resources for the project, selecting the RAD model was deemed the appropriate approach for this app development project.

The project group, which contributed to the RAD project, consisted of team members from the following subject groups: 1 project manager (AAS), 1 psychiatrist, 1 psychiatric nurse, 3 psychologists, and 1 patient with BPD (peer-worker) working in the psychiatric system. Furthermore, the project group included Skilled Workers with Advanced Tools (SWAT) members, that is, 1 software developer and 1 videographer. The clinicians were primarily responsible for selecting relevant evidence-based material to be integrated into the app. The peer employee contributed continuously with ideas and reviews regarding the app functionality and graphical user interface (GUI). The SWAT employees were responsible for the technical aspects of the RAD project, including video recordings of the evidence-based material and coding of the app. The project manager was responsible for the adherence to the project timeline.

**Figure 1:**
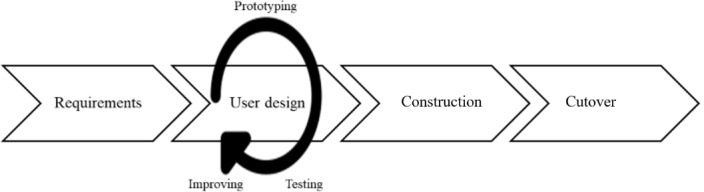
The 4 phases of rapid application development (RAD; adapted from Tan et al [14], which is published under Creative Commons Attribution 4.0 International License [15]).

The following constraints characterize the RAD process:

- The project group is relatively small, for example, around 6 persons.
- The project duration is relatively short, for example, a few months (<6 months). The RAD model aims to prioritize the time schedules agreed upon and, if needed, reduce the requirements to avoid increasing the deadline.
- The RAD project focuses on app development rather than process documentation.
- The RAD working process is iterative and incremental and includes 4 phases (Figure 1 [13, 14]).

### Requirements Phase

The requirements phase consists of the development of a high-level functional app specification, which is reviewed and agreed upon by all project group members. This phase includes joint requirements planning (JRP) workshops.

### User Design Phase

The user design phase consists of the development of design specifications for a functional app, which are reviewed and agreed upon by all project group members. This phase includes joint application design (JAD) workshops.

### Construction Phase

The construction phase consists of the implementation and coding of the app with contributions from all team members, including the SWAT members.

### Cutover Phase

In the cutover phase, the initial app system should be ready for implementation and testing in clinical practice.

### Ethical Considerations

The study focuses on the mobile application development process and does not require ethical approvals (EMN-2022-02740). Recruitment started after obtaining institutional review board approval (REG-123-2021). This study will be disseminated at scientific conferences.

## Results

All phases of the RAD model were implemented in the development of an app for people with BPD diagnosis waiting to commence psychotherapy.

### Requirements Phase

Several requirements were established at the beginning of the RAD project. The requirements were established across JRP workshops conducted with the team members, resulting in a backlog sheet encapsulating a list of requirements. During the cooperation and discussions in the JRP workshops, 7 requirements were selected from the backlog sheet and were equally prioritized to be implemented in the first release of the RAD project. The prioritization of requirements was achieved using the MoSCoW (must have, should have, could have, and won’t have [at this time]) prioritization technique [14. Due to the short time frame for the RAD project, only “must have” requirements were selected from the backlog sheet, resulting in 7 requirements.

First, the project managers decided that the app development time should be relatively short (within 6 months). Second, the population target was patients with BPD waiting to commence psychotherapy. Third, the mobile app should only contain evidence-based video themes targeting patients with BPD waiting to commence psychotherapy and themes already known by clinicians through their daily work. Fourth, the first theme (“Theme 1: Diagnostic criteria for BPD diagnosis”) should be interactive to increase usability. The other themes were not chosen to be interactive, as the project managers and SWAT team members agreed that it would be time-consuming and not achievable within the required timeframe (<6 months). Fifth, the themes should be presented or constructed by health workers (psychiatrists, mental health nurses, or psychologists) to ensure the evidence information provided through the themes. Sixth, the team member should primarily be locally affiliated (in-house development) to minimize costs, and a specific project budget was agreed on. Seventh, data security should be ensured.

### User Design Phase

The user design was an iterative process and included several JAD workshops with the team members. During the JAD workshops, the MoSCoW model was again used to prioritize the design aspects of the app. The team members decided and agreed on 4 main aspects of the user design. First, the mobile app should have an introduction page explaining the rationale of the app. Second, the mobile app should have a themes page presenting a list of all themes, and each theme should be presented through a video to increase the app’s usability. Third, the third page should contain contact information for relevant psychiatric institutions in Denmark providing support for patients with BPD. Fourth, the app should be user-friendly and not have unnecessary functions or elements.

During the user design phase, the project group discussed whether it was crucial to implement an introduction page. The group agreed it was necessary, considering the mentally vulnerable audience the app is intended for. However, the group also decided that the text on the introduction page should be concise, accurately reflecting the rationale behind the app. A telephone number was also included, allowing patients to call and obtain further information about the app and its purpose.

### Construction Phase

This phase covered a comprehensive plan for cooperation between team members. Each clinician was assigned a specific theme within the app and was responsible for drafting the first version of a video theme manuscript. The videographer was responsible for booking meetings with the individual clinicians and recording the videos with the clinicians in the individual psychiatric departments. Furthermore, the videographer instructed each clinician how to present each theme before video recording to ensure uniformity and enhance the video production of the themes. The close collaboration between the videographer and clinicians resulted in faster video production, thus optimizing the general development process. Content for the video themes was reviewed by an expert group of clinicians with experience in treating people with BPD to ensure the material was clear and concise and had a degree of conformity to facilitate overall interaction with the app. The programmer (SWAT team member) was employed full-time for this project and was in ongoing dialogue with the videographer and other participants. The focus was primarily on functions and GUI and the deployment of the videos within the app. The participants were iterative, contributing suggestions to improve the app. The coding of the functionalities and GUI took place iteratively and consisted of (1) unit tests, (2) integration tests, (3) system tests, and (4) acceptance tests [16]. Each test was only performed when the previous test had been implemented successfully.

New functionality or GUI was coded and tested independently as individual units (unit test). The newly developed units were then combined and tested with the existing units to ensure the compatibility of the newly developed units (integration test). The entire system, including the latest developed units (codes) was tested to assess its functionality and performance within the whole system (system test). Potential end users of the app were presented with various app mock-ups. The mock-ups enabled the end users to provide feedback regarding the app design and functionality at an early stage and before the app’s cutover phase. By releasing small functional releases, the development team got feedback from the end users by offering the users the opportunity to test the newly released designs and functionality (acceptance test).

**Figure 2:**
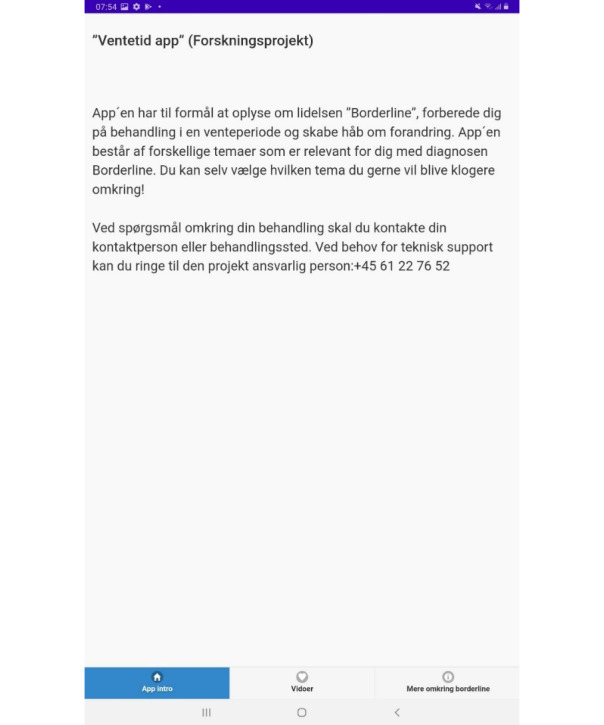
Home and introduction page. Translation: "Waiting Time App" (Research Project) The app aims to provide information about the condition "Borderline Personality Disorder," prepare you for treatment during a waiting period, and create hope for changes. The app consists of various video themes relevant for you with the diagnosis of Borderline Personality Disorder. You can choose which video theme you would like to explore further! For questions regarding your treatment, please reach out to your designated contact person or treatment facility. If you require technical support, you can contact the project coordinator at +45 61 22 76 52.

**Figure 3:**
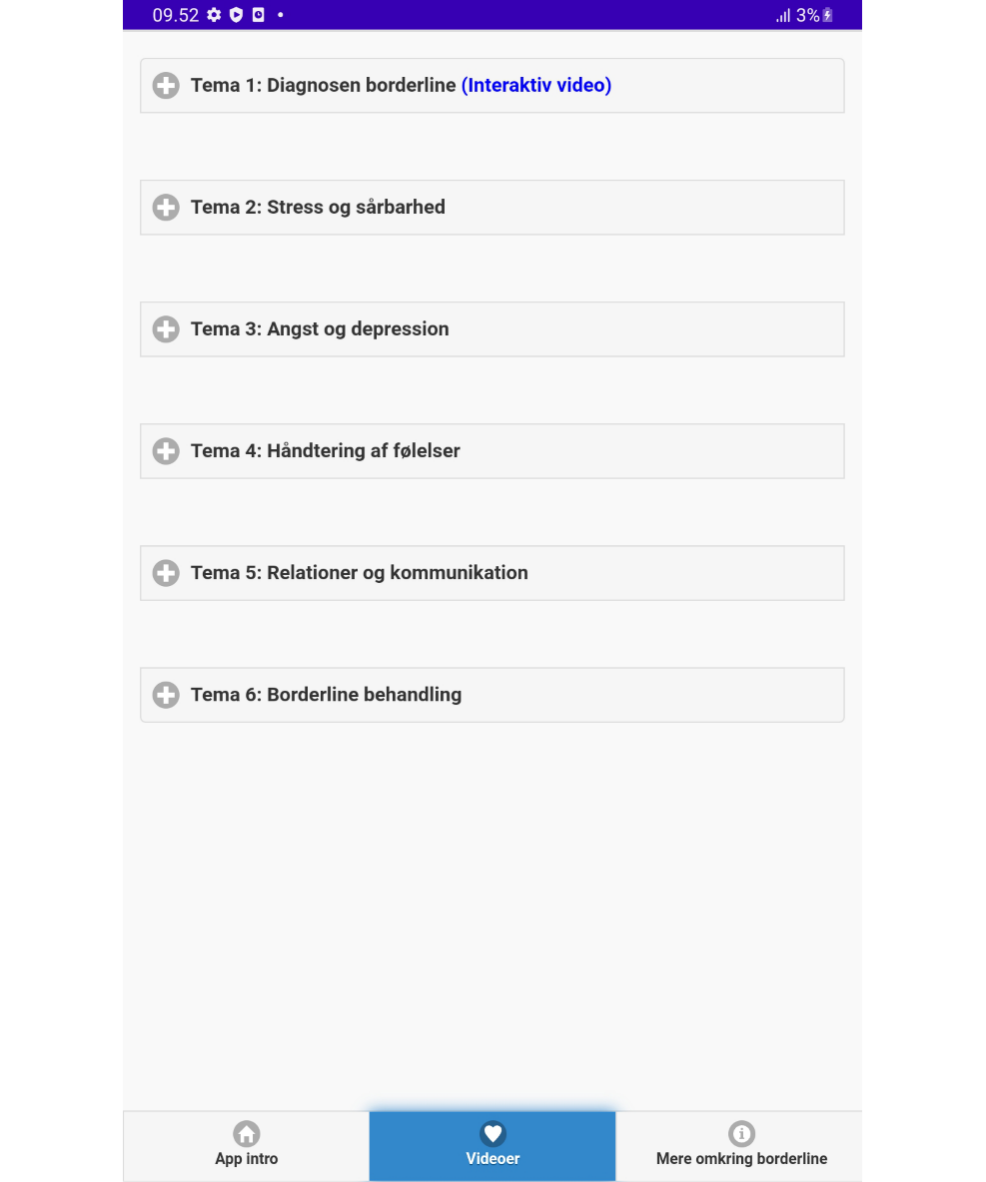
Video themes page. Translation: Theme 1: Borderline Diagnosis (Interactive Video) Theme 2: Stress and Vulnerability Theme 3: Anxiety and Depression Theme 4: Managing Emotions Theme 5: Relationships and Communication Theme 6: Borderline Treatment.

**Figure 4:**
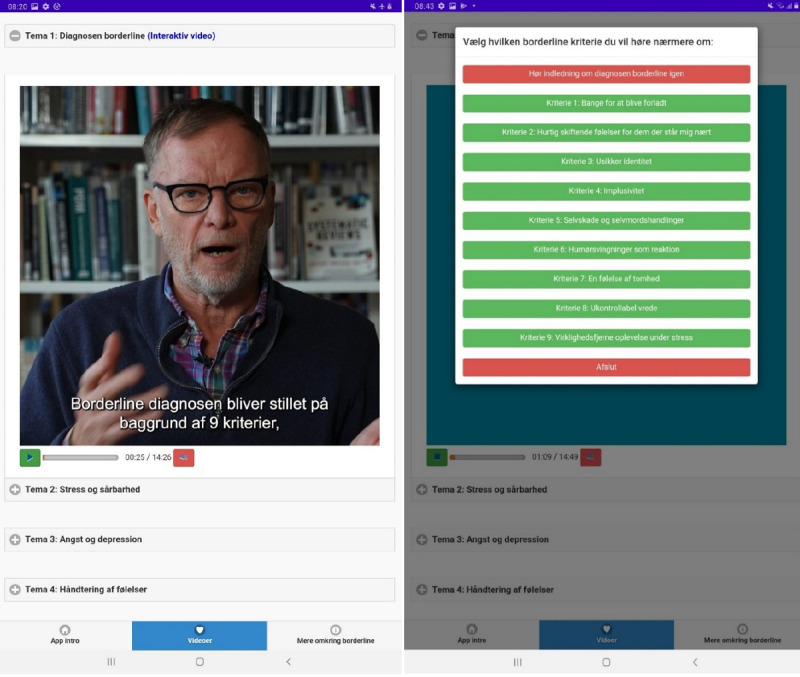
Video theme 1 ("Borderline diagnosis") with multiple subthemes according to the 9 criteria for Borderline diagnosis. Translation: Criterion 1: Frantic efforts to avoid real or imagined abandonment Criterion 2: A pattern of unstable and intense interpersonal relationships Criterion 3: Identity disturbance Criterion 4: Impulsivity Criterion 5: Self-mutilating and suicidal behavior Criterion 6: Affective instability Criterion 7: Feelings of emptiness Criterion 8: Difficulty controlling anger Criterion 9: Dissociative experiences under stress.

**Figure 5:**
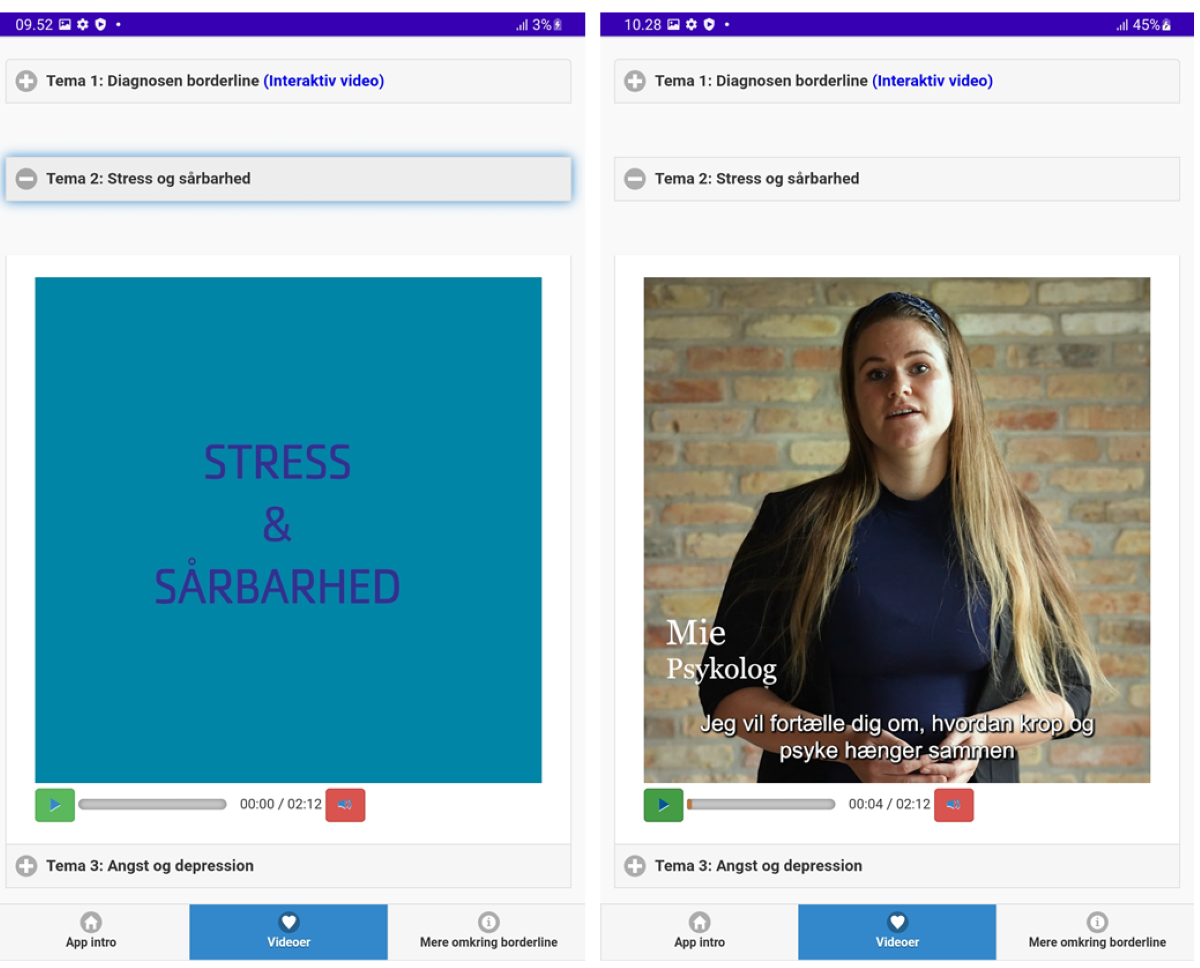
Video theme 2 ("stress and vulnerability"). Translation: Mie, Psychologist: I will now tell you about how the body and mind are interconnected.

**Figure 6:**
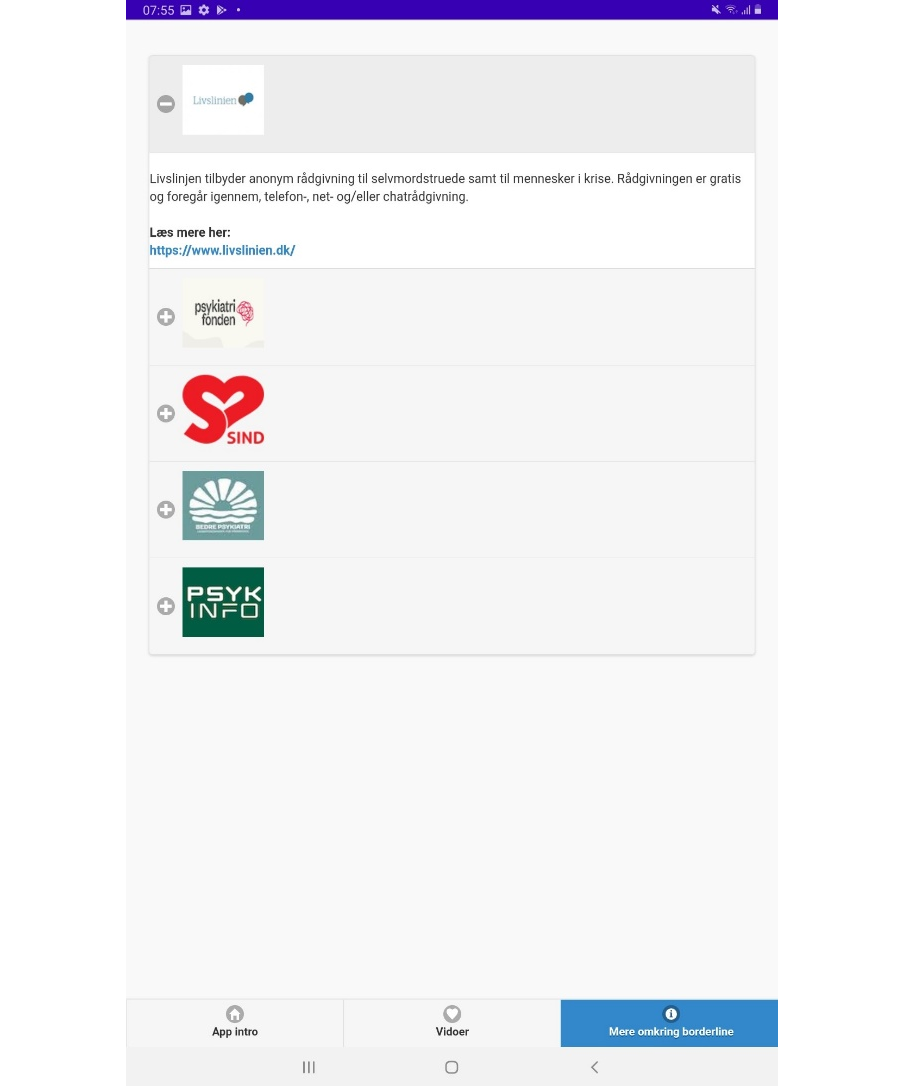
Support page. Translation: "Livslinien" provides anonymous counseling for those at risk of suicide and individuals in crisis. The counseling is free of charge and is conducted through telephone, online, and chat support. Learn more here: https://www.livslinien.dk/.

Several strategies were applied during the construction phase to ensure data security. First, tablets supplied for the patients during the cutover phase were secured with access control that the end users could not turn off. Second, the Android tablets used by patients were pre-encrypted by the manufacturer, which has been a requirement since the Android 10 release, as Google has required it from the manufacturers. Third, active data (“Click on themes/buttons”) that were collected were anonymized. The final app applied during the cutover phase is shown in Figures 2-6.

### Cutover Phase

Before the system was released and tested in clinical practice (“beta tests”), the system went through several alpha tests, that is, tested internally by the project group [17]. Both black-box (“external working”) and white-box (“internal working”) testing techniques were applied during the alpha test to identify and eliminate bugs before it was released for the patients in clinical practice. A group of 5 participants who were diagnosed with BPD and waiting to begin psychotherapy tested the mobile app for 7 days (beta tests). Informed consent was obtained from the participants. Participants were recruited from 2 outpatient clinics. Participants were recently diagnosed with BPD and are currently on a waiting list to commence treatment. The purpose of the beta tests was to examine how patients perceived the use of the app while waiting for treatment. Participants provided feedback about the positive or negative effects of interaction with the app during this time. All participants received a tablet with the mobile app installed. After completing a 1-week test, participants were invited to complete a follow-up interview and customized questionnaire regarding their experience with the app.

Results from the interview and questionnaire indicated that participants expressed high engagement with the app during the trial period. This feedback was supported by the multiple active data captured from the app. Participants described the app format as preferable to the paper form earlier used in clinical practice. Additionally, patients also described the use of interactive video (theme 1) as more user-friendly and interactive than the other video themes (static video themes). Moreover, participants highlighted that the app was not only used by themselves but also by their family members and relatives.

Participants also provided feedback about potential improvements to the app. First, they hoped the future app would integrate more interactive video themes. Second, they recommended additional video themes, for example, videos with “former” patients with BPD describing how they experienced and managed the waiting time for initiating treatment. Finally, some participants suggested embedding the app into a web app would increase accessibility to evidence-based video themes.

## Discussion

### Principal Findings

This study investigated how RAD could be conducted within an outpatient psychiatric setting by using “in-house” expertise from researchers, clinicians, patients, software developers, and video graphs. Using the RAD model, it was possible to develop a mobile app for patients with BDP on a waiting list to commence psychotherapy. While clinical staff were involved in the development of the app, the model ensured that there was minimal disruption on clinicians’ daily work. The model reused or used existing material (textual form) regarding BPD diagnosis and management that was currently used in clinical practice. This “reuse of resources” reduced the time clinicians needed to spend developing material for the videos and themes contained in the mobile app. Furthermore, involving patients and clinical experts during RAD ensured that the mobile app reflected the needs of people with BPD while waiting to commence psychotherapy. Overall, the process from development to implementation in the clinical practice was streamlined by applying the RAD model. Reusing existing evidence-based information material currently used by clinicians facilitated RAD. As clinicians were familiar with the evidence-based content material, there was no need to create new material or information for each video theme, decreasing the time and resources spent on the development process. By carefully selecting relevant experts and clinicians to contribute to the development process, ensured that the workload during the RAD project was evenly distributed across team members (videographers, researchers, clinicians, patients, and software developers). While the interactive video theme (“Theme 1: Diagnostic criteria for BPD diagnosis”) was time-consuming and dependent on an iterative cooperation process between the clinicians, videographers, and SWAT members, it did provide a unique format to provide evidence-based information.

Few studies have used RAD models for the development of apps within the health care system. A study by Tan et al [14], using RAD, developed an app during the coronavirus pandemic to remotely monitor and provide mental health care to patients with COVID-19. Consistent with our findings, the authors also emphasize that RAD models facilitated the app’s development cost-effectively, rapidly, and with high quality. Another development study, conducted by Ongadi et al [18], also used RAD to develop an app for detecting HIV drug resistance mutations and treatment at the point of care. In line with our results, the authors also emphasized that RAD facilitated engagement between stakeholders (patients, clinicians, and app developers) and that developing apps using RAD models was clinically suitable.

The RAD model also allowed for relevant adaptations. After the production of the first versions of the video themes (first iteration), it was clear that each video theme needed subtitles in order to increase the material to a wider audience (eg, people that were deaf or hard of hearing patients or those people who preferred to process information visually). As the clinicians had written manuscripts for each theme, it was relatively easy to use the manuscript to integrate subtitles in the videos.

The most significant challenges during the RAD project were time and resource management. Several approaches were taken early in the project to ensure that the deadline of 6 months was met and necessary resources were available to launch the first release in clinical practice. First, a project manager ensured the project was drafted and was responsible for planning, leading, and implementing the RAD project. Second, the project group defined a clear purpose for the app, and the requirements were well-defined by applying the MoSCoW model early in the project. Third, the project was divided into small, manageable parts to streamline the RAD. For example, each clinician was responsible for a video theme, and the videographer was responsible for coordinating a meeting with the clinicians to record the video themes, which made it feasible to produce multiple evidence-based videos within a manageable time frame.

### Strength and Limitations

This RAD model had several strengths. First, as it focused on in-house expertise (all team members were employed within Region Zealand Psychiatry, Denmark), this reduced the time, administration, and cost of employing external partners. Second, the use of existing evidence-based material to develop material for each video theme increased the development efficiency and reduced costs. Using input from clinicians also increased the validity and trustworthiness of the material presented. Third, the inclusion of fully dedicated team members (patients, clinicians, SWAT members, and researchers) at the initial phases of the RAD process facilitated rapid, high-quality (evidence-based), and low-cost app development. Finally, a significant advantage of applying RAD compared to other software development methods is that RAD focuses on a short iteration time of the complete, moderately sized user-stakeholder path, thus ensuring a short development time for delivering the first version of the mobile app. This advantage is not found in the alternative Scrum or Agile development methods.

The app and the RAD model also had several limitations. First, the present app is targeting a Danish patient group, thus limiting its generalizability and accessibility to a wider audience. With the objective of increasing the accessibility of the mobile app, it would be reasonably uncomplicated to translate it from Danish into another language. However, this process would require producing new videos and modifying the text in the app to align with new languages. The functionality and design of the app can still be reused. Second, the cutover phase (testing the app in practice) involved a small number of participants over a short time period with limited feedback. A more comprehensive evaluation involving a larger number of end users is needed. Third, software developed using RAD can lack breadth and depth [12]. Thus, one could use the RAD approach within psychiatric services as the first step to facilitating the in-house development of an evidence-based mental health app, followed by more extensive development and evaluation if required. Fourth, a prerequisite for RAD to function optimally is this model requires a small, experienced team with the necessary knowledge and skills, which may not be present in all mental health settings. While the RAD model is seen as an approach to facilitate the development, implementation, and evaluation of digital solutions in clinical settings, a range of human-computer factors need to be considered. Organizational issues including the organizations’ readiness for change, technological infrastructure, and digital literacy of end users are central to the uptake and impact of a digital solution [19]. Successful implementation also requires considering the specific characteristics, needs, and behaviors of the end users. It has been suggested that qualitative studies can also provide important contextual information and process dynamics to provide a deeper understanding of the factors that influence engagement [20].

### Clinical and Research Implications

There are several clinical and research implications of using the RAD model. First, there is a short timeframe from prototype to implementation or evaluation. This reduced time period means that apps can be implemented within a clinical setting quickly. Thus, a useful digital solution can be accessed by service users within the clinic setting and potentially provide benefits of reducing distress and promoting engagement or readiness for treatment. As this model allows for regular adjustments, it can be adjusted to reflect the current needs of a particular population.

As the RAD approach is a generic model, it also has the possibility to be applied in the development of mobile apps for a range of mental health problems. This flexibility in the model could be useful in psychiatric treatment where settings and the needs of service users can vary while acknowledging the model also requires a number of organizational conditions to be fulfilled.

The RAD model has also implications for research implementation as it can facilitate the integration of new digital solutions into clinical practice. The delay in implementing new approaches to routine care is recognized as one of the biggest challenges in research [21]. Additionally, this more rapid process promoted by RAD can help ensure that useful digital interventions can reach service users before they become outdated or irrelevant.

### Conclusions

This study demonstrates how RAD could be applied within a psychiatric outpatient service for developing an evidence-based mobile app for patients with BPD on a waitlist to commence psychotherapy. The RAD approach facilitated in-house development, using team members’ expert knowledge and skills working within the psychiatric outpatient services. The result was a clinically relevant technological solution that was able to be accessed by service users within a short timeframe**.** While recognizing the need for further studies to demonstrate the efficacy and effectiveness of mobile apps for BPD, this development study shows promise in addressing the unmet needs of waitlisted patients with BPD.

## Abbreviations

BPD: borderline personality disorder
GUI: graphical user interface
JAD: joint application design
JRP: joint requirements planning
RAD: rapid application development
SWAT: skilled workers with advanced tools
MoSCoW: must have, should have, could have, and won’t have (at this time)
